# “I'm a Referee, Not a Female Referee”: The Experiences of Women Involved in Football as Coaches and Referees

**DOI:** 10.3389/fspor.2021.789321

**Published:** 2022-01-31

**Authors:** Scarlett Drury, Annette Stride, Hayley Fitzgerald, Nia Hyett-Allen, Laura Pylypiuk, Jodie Whitford-Stark

**Affiliations:** Carnegie School of Sport, Leeds Beckett University, Leeds, United Kingdom

**Keywords:** football, women, feminism, coaching, refereeing

## Abstract

The development of the Women's Super League (WSL) in English football, increased media coverage of the game, and an expansion of grassroots opportunities indicate a bright future for women and girls who want to play. Yet this vision must be tempered against compelling evidence of deep rooted and enduring gender inequalities within the game. This is the case for both players, and women who undertake non-playing roles, which is reflected in the relatively low numbers of women coaches and referees. Whilst The Football Association (The FA) has signalled addressing these inequalities as a key priority, critics argue that such efforts amount to superficial and limited efforts to support meaningful change. This paper departs from a concern with *playing* the game and responds to calls for more research to explore the experiences of women involved in football in non-playing roles. More specifically, it focuses on women coaches and referees, and addresses the following question: how do women in positions of power in football negotiate their place in what remains a distinctly male-dominated profession? In addressing this question, we take a theoretical position located at the nexus between radical and post-structural feminism, acknowledging the significance of structural power relations and individual agency in shaping daily lived social realities. Data were generated from interviews with 14 women coaches and 10 women referees. These interviews explored the structure and culture of the game and its impact on women's experiences of men's and women's competitive and grassroots football. Through a rigorous process of thematic analysis, three themes were identified: gendered entry into football careers; reinforcement of women's difference on the football field; and coping strategies for remaining in the game. Centralising the women's voices in this research highlights the insidious and persistent nature of gendered microaggressions, the sexism of football culture, and the ways in which these women negotiate this masculine terrain in their pursuit of being coaches and referees.

“*Andy Gray and Richard Keys hauled off air for sexist comments”* (The Guardian, 24 January, 2011)

“*Crystal Palace Women goalkeeper accuses clubs of ignoring FA protocols after she was subjected to sexist abuse”* (The Telegraph, 16 January, 2020)

“*Football manager demands ban on women referees”* (The Guardian, 12 November, 2006)

“*Richard Scudamore sexism scandal intensifies as conspirator in sexist emails investigated by own law firm”* (The Telegraph, 16 May, 2014)

“*Soccer chief's plan to boost women's game? Hotpants”* (The Guardian, 16 January, 2004)

“*Women in Football survey a damning indictment of sexism in the workplace”* (HRreview, 11 March, 2014)

“*Clattenburg criticised for claim female referees must pick career or children”* (The Telegraph, 1 October, 2021)

## Introduction

It is widely documented that women's football has experienced a surge in popularity over the past 20 years (Pielichaty, 2015; Bell, 2019; Clarkson et al., 2020). The development of the Women's Super League (WSL) in England and increased media coverage of global competitions has gone some way toward recognising elite women players in popular culture (Woodhouse et al., 2019). Similarly, there has been an expansion of opportunities within schools and community settings through The Football Association (The FA) and Barclays' Girls' Football School Partnerships^1^, The FA's Shooting Stars^2^ initiative, and the emergence of the Wildcats centres^3^. The future seems bright for women who want to play football. Yet as the headlines above attest, sexism, exclusion, and discrimination remain ever present within the game. Indeed, the media continues to serve as a conduit for imparting subtle gendered messages that trivialise women's athletic performance, objectify their bodies, and reproduce narrow binary definitions of femininity (Pfister, 2015; Black and Fielding-Lloyd, 2019). This is the case for women who *play* football but also touches those who take on non-playing roles such as officials, referees, and coaches. When we dig beneath these headlines there is compelling evidence confirming the presence of deep-rooted and enduring gender inequalities within football (Caudwell, 2011; Clarkson et al., 2019). For instance, there remains a lack of women in positions of leadership and management. Women are significantly underrepresented in football coaching roles; the ratio of male to female coaches in English women's football stands at 96:4 (UEFA., 2017; Clarkson et al., 2019; Sawiuk et al., 2021). This pattern of men taking on coaching and management roles in women's football is very rarely the case for women in men's football (Caudwell, 2011; Clarkson et al., 2019). A similar picture emerges in relation to refereeing. Whilst there was a reported 72% increase in qualified women referees between 2016 and 2020 (Stimpson, 2020), there is still a considerable absence of women in officiating roles, particularly in men's football. Rebecca Welch made national news in early 2021 for becoming the first woman to referee a men's match in the English Premier League (MacInnes, 2021), yet men account for 37% of referees in the Women's Super League (Stimpson, 2020). Of course, even without these statistics, for those of us who regularly watch football in our local parks it is clear when you look beyond those playing, that women are less visible within the myriad of roles that enable the game to flourish. At a strategic level The FA is aware of these inequalities, and in its most recent policies, (The Football Association, 2017) *Gameplan for Growth* (2017) and (The Football Association, 2020) *Inspiring Positive Change* (2020), outlines key priorities for increasing the number of women coaches and referees. These include: establishing a more diverse workforce; one that is well-trained, ambitious and valued; and where women feel confident and empowered. However, critics argue that such efforts reflect a superficial and largely tokenistic approach which remains limited in its potential to affect any meaningful change at structural or institutional levels of the game (Welford, 2011; Jones and Edwards, 2013; Sawiuk et al., 2021).

The gendered dynamics of non-playing roles described so far are clearly not restricted to football. There is a substantial body of literature that highlights the problems of unequal representation of women in leadership positions in sport more broadly (see Burton, 2015). Collectively, this work sheds light on the different ways in which women are excluded, devalued, and discriminated against in various leadership roles in sports organisations. Norman (2010, 2014); LaVoi and Dutove (2012), and Norman et al. (2018) work, for instance, attests to the challenges faced by women negotiating their place in sports coaching careers. Other studies point toward the “glass ceiling” encountered by women in sports management positions (Claringbould and Knoppers, 2012), the neoliberal approach taken by sports organisations with respect to gender equity (Hovden, 2015), and the resistance expressed by (male) boards of governors in sports organisations to embrace and pursue gender balance in sports governance (Knoppers et al., 2021). These examples expose solid foundations for the deeply embedded sexism identified by Fink (2016), that surreptitiously pervades the broader landscape of sport. Whilst football constitutes only one small and specific part of this landscape, we argue that it is perhaps one of the contexts in which inequitable gendered power dynamics are most profound. Football has a history of explicit structural exclusion of women (Williams, 2003) and plays a key role in producing and solidifying dominant notions of masculinity in contemporary popular culture (Cleland et al., 2020). This, coupled with its status and popularity in England and dominant presence in sports media, means that football offers a unique context in which to explore the gendered dynamics of leadership roles in sport.

With these overarching issues around inequality and discrimination in mind, in 2011 Caudwell (2011) called for a continued analysis of the gendered nature of football culture to highlight the forces that “operate to affirm and reaffirm men's and boys' ‘entitlement' to the game” (p. 1). Over the last two decades a number of notable studies have uncovered the challenges faced by women and girls in football (e.g., Scraton et al., 1999; Clark and Paechter, 2007; Ratna, 2011; Stride and Fitzgerald, 2011; Welford, 2011; Pielichaty, 2015). This research has focused predominantly on women and girls' opportunities to *play* football. In 2014, however, Norman (2014) noted that there were very few studies exploring women in sports coaching and leadership roles that offer a critical perspective on the institutional and organisational structures of sport. Although several authors have since added to this area of scholarship, as we highlight in the next section, there still remains a comparative lack of insight into gendered experiences of women involved in football as coaches and referees. Mindful of both Caudwell (2011) and Norman (2014) expositions, this paper seeks to address the following question: how do women in positions of power in football negotiate their place in what remains a distinctly male-dominated profession? In doing so, we hope to offer insights that can be used by football governing bodies, organisations and clubs to inform the future development of coaching and refereeing opportunities for women. We draw on data generated by three research projects exploring the experiences of women football coaches and referees. Although each of the projects focused on slightly different football contexts, we recognised synergies in the theoretical and methodological lenses adopted and the findings which, in combination, offered concerning evidence of the widespread and pervasive nature of gender discrimination in football.

## Literature Review

Over the last two decades, the marginalisation of women in football has been extensively documented in academic scholarship (e.g., Scraton et al., 1999; Caudwell, 2003; Williams, 2003, 2007, 2019; Bell, 2019; Clarkson et al., 2019; Cleland et al., 2020). Historical accounts of women's involvement in the game (e.g., Lopez, 1997; Pfister et al., 2002; Williams, 2003, 2019; Williams and Hess, 2015) have traced the origins of this oppression and demonstrated how key milestones, such as the institutional banning of women from competition on FA affiliated grounds, have contributed toward the challenges faced by women footballers in gaining recognition in UK society today. These accounts provide insights into the deeply-rooted discourse that positions football in the UK as a distinctly masculine preserve. Set against this backdrop, the advances made by women in football in recent years might seem impressive. For example, over the last three decades, participation in the game by women and girls has seen unprecedented growth (Clarkson et al., 2020). Football is now one of the most popular sports regularly undertaken by women, with a record number of women's and girls' teams competing in grassroots leagues and at the highest echelons of the game (Bell, 2019).

However, Scraton et al. (1999) argued for the need to move beyond a restrictive focus on participation rates as a means of determining the relative progress of women in football. They cautioned that despite more women gaining access to football, the taken for granted connexions between football and masculinity remain problematic for women involved in the game. Furthering this agenda, subsequent studies have explored the structural, institutional, and discursive forces that challenge women's advancement in football. For example, Swain (2000) and Clark and Paechter (2007) highlight how girls' access to informal football opportunities at school are limited by the spatial monopolisation of playgrounds by young boys. Jeans and Kay (2007); Jeanes (2011), and Pielichaty (2015) work explores young girls' experiences of negotiating the contradictions between “feminine” and footballer identities. Similar themes are explored amongst adult women footballers by Welford and Kay (2007) and Themen (2016), who uncover the tensions that arise between normative notions of femininity and the physicality required for football prowess. Cox and Thompson (2001); Caudwell (2003, 2007); Mennesson and Clement (2003), and Drury (2011) extend these discussions in their identification of the role of homophobia and heteronormativity in shaping women's football experiences. This work highlights the way in which gender discrimination is underscored by heteronormative ideas that equate women's entry into football with the embodiment of “deviant” sexual identities. This leads to homophobia being used as a means of policing women's freedom to engage in playing football (Caudwell, 2003). One of the most important contributions made by these studies collectively is the examination of the discursive tensions between femininity and football, and the subsequent contradictions associated with players' abilities to negotiate their footballer and gender identities. By competing in football, women transgress the boundaries traditionally associated with “acceptable” femininity. Yet in doing so, they help to redefine taken-for-granted notions of football as a male space. Research has also explored the intersections of gender and other axes of social difference in the context of football. King (2007), Ratna (2011), and Ahmad (2011), for example, document the ways in which discourses of gender and ethnicity influence women's negotiation of their place within football. Similarly, Stride and Fitzgerald (2011) consider how a group of learning disabled girls challenge expectations around gender and ability in football. Moving debate away from women's experiences, a small number of studies have also interrogated the role of the media in shaping popular understandings of women's place within the game. This research has been instrumental in highlighting not only the lack of attention given to elite women players compared to their male counterparts (e.g., Bell, 2019), but also in demonstrating how subtle gendered messages are imparted on media consumers that trivialise women's athletic performance, objectify their bodies, and reproduce narrow binary definitions of femininity (Pfister, 2015; Black and Fielding-Lloyd, 2019).

Whilst earlier scholarship on women in football focused predominantly on women and girls' opportunities to *play* the game, a small number of more recent studies have considered women's experiences of coaching and officiating football. Collectively, this research draws attention to the challenges associated with women gaining meaningful entry into an environment that epitomises hegemonic male dominance. As Jones and Edwards (2013) note, it is important to distinguish between playing and non-playing roles when exploring the gender dynamics in football. Whilst advances have been made toward eradicating essentialist myths about women's biological inadequacy for playing football, progress has been comparatively slower in terms of challenging unfounded assumptions about women's alleged incapability to fulfil leadership or decision making roles (Welford, 2011; Jones and Edwards, 2013; Reid and Dallaire, 2019). This is reflected in the relatively low numbers of women currently operating in coaching and officiating roles in football (Clarkson et al., 2019; Reid and Dallaire, 2019), and provides another reason to be critical of claims of near equality in the sport (Graham et al., 2013).

The coach education process has been identified as a particular concern and remains an overwhelmingly male dominated arena, with coach educator roles largely taken up by men (Fielding-Lloyd and Meân, 2008; Schlesinger and Weigelt-Schlesinger, 2012; Norman, 2014; Lewis et al., 2018; Fasting et al., 2019). This results in women feeling “unwelcome” in the coach education environment, which is further exacerbated by the behaviours and language used by male coach educators, who favour traits they perceive to be associated with masculinity (Fielding-Lloyd and Meân, 2008, 2011; Lewis et al., 2018; Sawiuk et al., 2021). Sawiuk et al. (2021), in their study of women undertaking The FA's UEFA A licence coaching programme, also note how the inappropriate language and behaviour of other (men) coach learners often goes unchallenged, further contributing to the androcentric nature of the coaching pedagogical environment. This androcentricity is further accentuated in a lack of coach education material relating to women's football, constant references to the men's game, and the coach educator's lack of knowledge regarding women's football. Schlesinger and Weigelt-Schlesinger (2012) argue that male coach educators have gender-specific expectations of women's coaching capabilities, which they perceive to be less compatible with football compared to their male counterparts. Exploring the experiences of women in football coach education, Fielding-Lloyd and Meân (2008, 2011, 2016) argue that trainee women football coaches feel more “under pressure” to “prove themselves” than their male equivalents. Their findings demonstrate that men's gendered identities afford them automatic and unquestioned access to the social dynamics of the coach education space. Women, on the other hand, have to earn this through their performance of appropriate levels of football knowledge and skill, and these expectations often go far beyond the level of performance required by men to gain the same credibility (Fielding-Lloyd and Meân, 2011, 2016).

Other studies reveal that these gendered dynamics extend beyond coach education. Fasting and Pfister (2000) note that the likelihood of men occupying coaching positions increases significantly toward the more elite end of the sports performance spectrum. Fasting et al. (2019) indicate that this could be attributed to the influence of social capital in the elite coach recruitment process. They suggest that senior coaches are often headhunted via social and professional networks, which tend to be dominated by men. Graham et al. (2013) argue that in order for women to be successful in higher profile coaching positions, they often have to gain higher levels of qualifications than those expected of their male counterparts to be considered serious candidates. These findings mirror Theberge's (1993) claim that women's “token” status in coaching results in a heightened and specific “pressure to perform” compared to men coaches. Coaching experience also appears to be valued in very gendered ways, with experience of coaching men's football carrying more “credibility” than coaching women's teams (Graham et al., 2013; Baldwin and Vallance, 2016). This is something that is applied to both male and female coaches, despite the social expectation that women will not have coaching responsibilities within men's clubs (Clarkson et al., 2019). Importantly, this reinforces both the status of men's football as “authentic” as well as the perceived legitimacy and normalcy of men in powerful football roles.

Echoing the literature on women in football coaching, a similar picture emerges from research exploring women football referees. Jones and Edwards (2013), Forbes et al. (2015), and Reid and Dallaire (2019) all draw attention to the conspicuous absence of women in football officiating roles, suggesting that gender disparities in officiating are perhaps even more profound than those found in coaching. Interestingly, whilst Jones and Edwards (2013) and Forbes et al. (2015) studies both focus on the inextricably masculine discourse of the UK football context, Reid and Dallaire (2019) findings show that this gender dynamic is still heavily present in the context of Canadian soccer, which is known to be much more “gender-neutral” in terms of expectations around who should play the sport. This lack of representation of women in football refereeing presents a number of challenges. For Reid and Dallaire (2019), women officials occupy a paradoxical position, in which their scarcity means they are viewed as a spectacle and a focus of attention, yet they are simultaneously sidelined and discredited. All three of these papers attest to the gendered assumptions that circulate around the perceived incompetence of women to fulfil officiating roles. It is regularly assumed that they lack the knowledge required to make correct decisions, that they fail to embody the assertive dispositions needed to control the game, and that they do not possess the physical capabilities to “keep up” with play, particularly in the elite men's game. Jones and Edwards (2013) argue that these assumptions stem largely from unfounded biologically deterministic claims of sex difference that circulate within the football profession. Like their coaching counterparts, women referees face the continued challenge of having to “prove themselves” in order to gain respect and recognition (Forbes et al., 2015). For many women referees, the process of gaining acceptance presents another interesting paradox; that is, the pressure of “reconciling” femininity and football officiating, which are socially deemed mutually exclusive. Forbes et al. (2015) argue that whilst women are more accepted when they “perform” a football identity in traditionally established ways, they are simultaneously condemned for their failure to appropriately perform normative femininity or criticised for “behaving like men” (Welford, 2011). This can lead to women officials experiencing victimisation often in the form of sexist and homophobic abuse (Jones and Edwards, 2013; Forbes et al., 2015).

It is clear from the existing body of literature that there is still much to do in challenging the inequitable gendered power relations that shape the sport. Whilst women have undoubtedly made significant inroads in terms of their involvement as players, the same cannot be said of women's involvement in non-playing decision making roles in football. We can assume that this is in part due to the greater ideological threat that women in positions of power pose to football's discursive status-quo. Women in these roles are uniquely positioned at the intersection of two distinctly male domains—football and leadership. Yet greater consideration of women's experiences of coaching and refereeing in football is needed in order to understand the complexities of the gendered dynamics that shape their presence in the game.

## Football and A Feminist Lens

Caudwell (2011) outlines the significance of feminist theorising to the study of gender and football culture. She highlights how liberal feminist struggles for gender equality, built around arguments for the sex-gender distinction, have been instrumental in securing women's and girls' access to opportunities to play football. Such activism has also resulted in significant changes to gender relations within football culture, with notable developments in the structure and governance of the women's game in recent decades (Caudwell, 2011; Jones and Edwards, 2013). Norman (2014) similarly argues for the need to adopt a feminist lens when exploring women's experiences of coaching. Critiquing earlier studies of the gender imbalance in the coaching profession, she argues that it is vital that scholars challenge the tendency to equate women's absence in leadership roles in sport with either their own alleged inadequacies, or with merely a lack of opportunities. Instead, analyses of the gender dynamics of coaching must attend to the socio-historical, structural and institutional contexts that have shaped women's marginalisation from sports leadership roles (Norman, 2014). In doing so, the focus of interrogation moves away from women themselves and toward the organisational and institutional practises that produce and perpetuate discrimination. Some of the studies on women in football coaching or refereeing reviewed so far have demonstrated the value of feminist theorising in understanding women's position in the sport (Jones and Edwards, 2013). For example, Clarkson et al. (2019) deploy Connell's conceptualisation of hegemonic masculinity to uncover the structural gendered inequalities present within football coaching. A similar theoretical approach is adopted by Lewis et al.'s (2020) in their exploration of gender-based violence in sport and coach education more broadly.

Our theoretical position is located at the nexus between radical and post-structural feminism. In early discussions of this paper, we recognised an overlap between the theoretical lenses that we typically adopt in our research. For Annette, this is about a concern for “middle ground” thinking, which recognises the significance of more structural approaches to understanding gendered power and women's subordination, whilst simultaneously acknowledging post-structural arguments for women as active agents in resisting and challenging power relations (Stride et al., 2019). Similarly, Scarlett's “feminist-queer” approach brings together the insights of radical feminist challenges to structural power relations of sport, whilst attending to the queer project of exploring the complex ways in which discourses of gender and sexuality are negotiated by individuals involved in sport at interactional levels (Drury, 2013). Our stance for this paper therefore reflects our combined endeavour to expose the various ways in which gender impacts women's involvement in football.

## Methodology

This paper presents the findings of three undergraduate research projects^4^ based in England that, taken together, offer unique insights into the ways that women involved in the delivery of football as coaches and referees experience their roles. Whilst each project had a similar focus, they differed in relation to the specific contexts explored. Project 1 (Jodie) centralised the experiences of five women football coaches working at different levels in both the men's and women's game—from those with FA level 1 coaching qualifications involved in facilitating grassroots youth football, to those completing their UEFA B Licence and working toward the elite level. Project 2 (Laura) focused on nine women coaches' experiences of participating in “women only” and mixed sex coaching courses. Project 3 (Nia) moved away from coaching, instead focusing on the experiences of 10 women football referees, working in both women's and men's football. Similar to the participants in Project 1, these women represented a broad spectrum in terms of their level of qualifications and experiences, with a mixture of elite and grassroots referees. And, like the participants in Project 2 they came from both the conventional mixed sex referee education pathway as well as a relatively new “women only” fast-track programme. All three of the projects involved researching women working in both women's and men's competitive football.

These studies share further similarities in the methodological approaches utilised. All adopted a critical interpretivist lens and used semi-structured in-depth interviews, recognising the value they bring in centralising the voices of the women participants, illuminating heterogeneity within the group, and unearthing similarities in experiences. Nia, Laura, and Jodie each developed their interview schedules in consultation with their supervisor (either Scarlett or Annette). These discussions helped to refine their schedules, taking into consideration past studies and each project's overall aim. As such, the final versions shared similarities in their focus on women's socialisation into the game, their key influences in their coaching/referee pathways, the challenges encountered, and the ways these were navigated and negotiated. All three projects adopted a purposive and convenience sampling technique using existing networks. Nia, Laura, and Jodie were heavily involved in football in various capacities at the time of the studies—Nia (Project 3) as a referee, Laura (Project 2) as a coach and Jodie (Project 1) as a player and coach. This enabled them to identify women coaches and referees with differing years and levels of experience in their respective roles, from different age groups, working across a number of counties in England^5^. For each project, interviews took place once informed consent had been given by the participants. In total, 24 interviews took place at a time and place convenient to both the interviewer and research participant, with each interview typically lasting between 30 and 45 min. These were then transcribed verbatim, and participants were assigned pseudonyms to maintain their anonymity. The gender and footballing identities and experiences of Nia, Laura, and Jodie were invaluable in not only facilitating data collection but in providing unique vantage points from which to analyse the data.

As the lead researchers of the three projects, Nia, Laura, and Jodie each analysed their data sets guided by the six phases of thematic analysis: data familiarisation; code generation; theme searching; reviewing themes; defining themes; and report production (Braun and Clarke, 2006). During this process, the researchers met with either Scarlett or Annette independently, who acted as critical friends in interrogating the initial identification of patterns of meaning, codes, and themes. These discussions encouraged reflexivity whereby Nia, Laura, and Jodie reflected on their positionality within football and research. More specifically, they considered how their history, roles, and experiences of the game interlink with their theoretical understandings and knowledge of past literature influencing their researcher judgement at each phase. Thus, data analysis became a recursive process moving backwards and forwards through the six stages, before finally defining a set of themes for each study. Guided in part by the study's overall research questions, prior literature, and theoretical frameworks, the lead researcher on each project moved between an inductive and deductive approach to data analysis. Whilst the themes identified were strongly linked to the data, “researchers cannot free themselves of their theoretical and epistemological commitments, and data are not coded in an epistemological vacuum” (Braun and Clarke, 2006, p. 84). Our discussions around researcher reflexivity and positionality were key to interrogating how a more deductive, theoretical approach influenced analysis.

It was at the point that the lead researchers had defined themes, that Scarlett and Annette began to recognise the similarities across the data sets through informal discussions. This prompted them to consider the significance of these data sets in combination. In so doing, Scarlett and Annette were mindful of the need to adopt a more latent approach to analysis to ensure the situatedness of the data within broader social discourses was captured. Whilst Nia, Laura, and Jodie had adopted a feminist perspective, their analysis at this point had taken a largely sematic approach which took participants' accounts at face value, and focused predominantly on identifying instances of inequality and discrimination. Scarlett and Annette were keen to move beyond this liberal feminist and relatively surface level analysis to begin to consider the significance of the structural, cultural, and material discursive conditions of these women's lives. Whilst they recognised the value of these women coaches' and referees' accounts per se, they were also interested in the meaning behind their accounts, how these had been shaped, and the resultant implications. More simply put, they were keen to address the question, “What is the overall storey the different themes reveal about the topic?” (Braun and Clarke 2006, p. 94). This paper reports on the themes from this latent approach: gendered entry into football careers; reinforcement of women's difference on the football field; and coping strategies for remaining in the game.

## “The Only Female Referee”—Gendered Entry Into Football Careers

All of the participants at various points throughout the interviews alluded to their “token” status (Theberge, 1993) as one of a minority of women referees or coaches working in football. For example, Clare discussed her experience as the only woman on both of the coach education courses she had completed. Similarly, Ella identified that she was the only woman with coaching responsibilities within her club, and Hattie described herself as “the only female refereeing locally.” However, it is important to point out that despite their minority status, all of the women spoke positively about the enjoyable and rewarding experiences brought about through their coaching and refereeing, and many had ambitions to achieve coaching and refereeing positions in the highest echelons of the game. Some also expressed their pride at being in a minority of positive role models for other girls and women with aspirations of a career in football:

I'm proud to be doing something different, not many females officiate so I like to be representing my gender (Clare, referee).

Whilst it is understandable and indeed laudable that women like Claire should feel eager to be a visible signifier of positive change, this perspective can also be interpreted as symptomatic of the broader gendered culture of football, as well as an increasingly neoliberal and postfeminist discourse that positions women as responsible for their own representation and liberation (Toffoletti, 2016). Sportswomen are regularly framed as ambassadors for women and girls in sport, in ways that are rarely expected of their male counterparts. Whilst many sportswomen are willingly compliant with this expectation (Norman, 2012; Dunn, 2016), this brings about an added level of responsibility and pressure to succeed, as we discuss later.

The interviews highlighted further challenges that were brought about by the women's minority status within a largely male dominated arena (Clarkson et al., 2019). For some, this involved practical or logistical issues related to accommodating women in an otherwise male domain:

Oh I think changing rooms was the first one. I got every single best quality broom cupboard or boiler room you can imagine. There was never a thought at all that clubs would need to accommodate women. I used to get things like, I'd turn up, particularly if I had two male neutral assistants, they would, the secretary or the club connexion, would look at the two assistants and ask “which one of you is the referee?” (Anne, referee).

For others, there was a sense that men did not know how to react to their presence. Hattie, a referee in men's Sunday league football, commented that players “might occasionally shout sir” when appealing to her to call a decision in their favour. More worryingly perhaps, the widespread perception that men were the “norm” in coaching and refereeing meant that women were offered few opportunities and were regularly overlooked in terms of the contributions they could make:

I was put with the younger age groups and I felt like that was because they felt that I didn't have the ability to coach the [boys'] under 14s. And I was very put off by that because in fact I was the highest qualified coach that they had. I took a back seat because men in that situation have a big ego about themselves... not all of them, because some of them didn't mind, but like a couple of them were assertive in saying ‘yeah I want the older boys' and my ability was overlooked (Ingrid, coach).

I'm a B licenced coach and Club X (men's) academy were screaming out for B licence coaches to take the boys and yet they've never ever approached, made an interest, or picked up the phone to enquire whether I fancy taking the … I don't know, under 8 boys, so that's never actually happened (Meg, coach).

When I was working for a club erm… the academy rang up and sort of asked for the most qualified coach and they said it was Maisie, she's just done her B licence and they said no we want a male (Maisie, coach).

For many of the participants, this meant that whilst they did experience some level of acceptance from men in similar positions within the game, there was an underlying feeling that this was conditional, and contingent upon them “proving themselves” against a distinctly male-defined norm. Like the participants from Fielding-Lloyd and Meân (2008, 2011, 2016) research, many spoke of having to “earn their place” amongst male coaches. Ella, for example, suggested she felt she had to “work twice as hard” as her male counterparts to establish herself as a credible and knowledgeable coach. Anne and Millie shared similar experiences:

If somebody measured me against a man, and said show me the skills in a particular area and if I didn't cut it up to the same scratch as the man, great. But I should not be measured on my gender alone. I've forgotten more about offside than most males ever know, so you know, why judge me on being a female whether I know the offside law or not. I've been a FIFA instructor for so many years, travelling the world, working on World Cups – of course I know what I'm talking about. I have to (Anne, referee).

The blokes were surprised to see a female on the course at first and, erm, there were some big characters in the group so I took a bit of a back seat. I think that changed when they saw that I could actually play football myself and that I was a good player (Millie, coach).

Many of the women felt that whilst some level of change was taking place with more women becoming involved in coaching and refereeing, this wasn't always helped by those responsible for governing the sport. Participants were critical of the dismissive nature of some of the men they encountered in decision making positions and those responsible for implementing development programmes for women referees or coaches (Welford, 2011). Anne's narrative was particularly illuminating in this sense. She argued that it was largely “men making these decisions,” some of whom were “pretty anti-women,” and that when a new strategy was implemented, they “either ignored it or couldn't be bothered.” Anne further alluded to the often tokenistic nature of strategies aimed at encouraging more women into refereeing and coaching in football.

I went to a County FA and a guy came down with all these leaflets he wanted to send out to get more women as assessors and mentors which I said was fantastic. I looked at the leaflet and every single image was a white male and I said ‘so how do you think a woman would look at that? Is she going to think that's for her?' Probably not. Make it relevant, target markets where women will be, [where they] will see [it] and will feel that it's for them. Make them feel it is a job that they can do.

As discussed earlier in this paper, at a strategic level concerted efforts have been made to initiate programmes that seek to support more women in leadership, refereeing and coaching roles. Despite these developments, it is striking to note that the material experiences of women within football have seen little change. In many respects, these accounts reflect those found in previous research; it might seem that there is nothing new here (Welford, 2011; Jones and Edwards, 2013; Forbes et al., 2015; Sawiuk et al., 2021). Yet importantly, this lack of change highlighted in our findings itself tells a bigger storey about the importance of understanding the ways in which strategic endeavours are re-negotiated within football clubs and teams; new policies may be introduced, but their rhetoric becomes easily lost in the ever-pervasive gender discourse of football.

## “Get Back in The Kitchen”—Reinforcing Women's Difference on The Football Field

Women coaches' and referees' minority status, and the subsequent repeated comparisons of women against male “norms” within the game, also appear to result in the constant discursive positioning of women as distinctly different from, and inferior to, men (Fielding-Lloyd and Meân, 2011; Welford, 2011; Jones and Edwards, 2013; Reid and Dallaire, 2019). This occurs in a number of different ways, yet the foundation for this perspective appears to be a fundamental belief that women's football is different from men's football. A number of participants indicated that “the male game” was widely perceived as “better” than “the women's game.” This involved the perception that male players were faster, stronger, fitter, and more skilful than women players.

People have the perception that women's [elite] football is the equivalent of a grassroots men's semi-pro team who train twice a week and you've got a game at the weekend. Where actually, we've got basically the same training load as a men's premier league team. Maybe we don't have the same facilities and the players don't get paid the same but it's the equivalent. And because men's football is all over the media, yeah, I'd say your average Joe would perceive men's as being better, when actually it's not (Maisie, coach).

Whilst many of the participants were keen to dismiss the idea that women's football was “lesser than” men's football and to assert the professionalism and athleticism required at the highest level in the women's game, it appeared that the rhetoric surrounding women's football as being different from men's football was at times uncritically reproduced by women coaches and officials themselves. This occurred to the extent that some participants described men's and women's football as “fundamentally different sports” (Maisie, coach), or “two completely different games” (Clare, referee). The rationale for this difference related to both a sense that the men's game was faster and more skilful, but also to the idea that the women's game was more “respectful.”

I think they're very different games. I feel like men's football is more fast paced football, and women's is a lot more slower and it's about actually passing the ball around (Millie, coach).

I think the play is different, I think the technical aspects of the game are different, the tactical side's different (Helen, coach).

They are two completely different games. I feel females have more respect for me, less likely to receive abuse or question my decisions. Male football is more challenging to referee as the game is a faster game and you are more likely to witness more controversial decisions and forceful tackles which require instant, confident judgements (Clare, referee).

I think male and female football is completely different, you coach it differently, the tactics are different, the environment, I think literally they are fundamentally two different sports (Maisie, coach).

In the context of coaching and refereeing, experience in men's football also appeared to carry greater value and prestige than experience within the women's game. Previous studies have highlighted this in relation to coaching (Graham et al., 2013; Baldwin and Vallance, 2016; Clarkson et al., 2019; Fasting et al., 2019), and in this research, this was also applicable to refereeing. Indeed, there was an assumption that to become a “better referee” you need to be involved in men's football:

I've followed the men's [pathway], started on the men's. It's a quicker pace, better fitness required for men, so then when you go into women's, it's fine. If you just do women's, you won't push yourself as hard. It might change if women's football becomes more... but in men's football there is a bigger and better outcome (Hattie).

This example adds credence to Fielding-Lloyd and Meân's (2008) argument, that separatist approaches to gender equity in sports organisations can be problematic in their tendency to reassert binary discourses that result in the othering of women. Such approaches also inevitably lead to the framing of women's pathways as “inferior” to the established (male) standard (Fielding-Lloyd and Meân, 2008). Whilst Fielding-Lloyd and Meân's (2008) commentary relates to separatist women-only coaching courses, our findings demonstrate that similar perceptions occur in relation to The FA's “Women's Game Pathway” for referees. Whilst not solely aimed at women referees, the marking of the pathway as explicitly associated with the “Women's Game” positions it, according to Hattie's account, as less challenging than the conventional (male) referee qualification route.

For some participants, there was also a belief that women and men brought different attributes and qualities to the game.

We bring in different skill sets. I think as a female we bring softer skills so we can diffuse a situation sometimes so that's a positive effect that we can have in a game (Jane, referee).

Based on previous research (Fasting and Pfister, 2000; Fielding-Lloyd and Meân, 2011), the perception that men and women have distinctly different traits is not uncommon in the football coaching profession, particularly among male coach educators. Yet wider research on sports leadership indicates that this “difference-oriented” position is increasingly being espoused by men in sports organisations as a rationale for greater involvement of women in senior positions, in instances where male support for women in leadership does occur (Kempe-Bergman et al., 2020). Like Fasting and Fasting and Pfister's (2000) study, it is apparent in our findings here that messages about gender difference are still internalised by women themselves. Despite this, the women were simultaneously critical of this “difference-oriented” perspective, and felt it was this way of thinking that was responsible for them being held up to greater scrutiny in relation to their knowledge, decisions and expertise. In particular, some of the referee participants felt that others were “waiting” for them to make an incorrect decision, and for any poor judgements on their part to be attributed to the fact that they are women.

High profile male referees get decisions wrong all the time but you never hear comments about their gender, you just hear that they made a mistake (Bea, referee).

There will always be someone who doesn't take gender into account but there will be others that will point out that I've got [it] wrong due to being a female (Hattie, referee).

This added level of scrutiny, coupled with the women's minority status in coaching and refereeing, posed further challenges that these women had to overcome in order to fulfil their roles (Forbes et al., 2015; Clarkson et al., 2019). Some of the referees expressed the nervousness they felt prior to a game, which stemmed from their uncertainty about the reactions of male players and coaches.

As I've just gone into open age I get most nervous just turning up and the perception they have of you. I know once I get started I can use my voice and signals to assert my authority but I find it trickiest when I get there (Sarah, referee).

I definitely think it's easier for men. When you walk onto the pitch and see it's a man there is a very different expectation of what the game is going to be like (Esme, referee).

The reactions of male players, coaches and officials toward the presence of women in coaching and refereeing roles were instrumental in further reinforcing the women's feelings of difference and otherness in football. Findings presented so far demonstrate that women often appear as an unexpected presence in coaching and refereeing roles, particularly in the context of the men's game. Participants commented that this led men to respond in a number of different ways when presented with women in football leadership roles. This was particularly profound for Anne who was one of the first women to officiate men's football at professional level:

I was a shock to everyone really. I don't think they ever really knew how to take [me] anyway, I think I shocked people everywhere I went really.

Somewhat predictably, one of the most common reactions of men discussed by the participants was the continued presence of sexist or homophobic comments that were made as a means of undermining their authority, or further highlighting their difference (Norman, 2010; Forbes et al., 2015). These ranged from the readily anticipated comments related to traditional gender roles, to the much more alarming overtly abusive sexist or homophobic denunciations.

Things like ‘don't you know the supermarket is open, you should be there doing the shopping', ‘why aren't you at home cooking your husband's meal' or ‘you should be pregnant at the kitchen sink' or the worst one was ‘I hope your children die of cancer' – that was from a spectator… I remember one game, an FA cup round and I walked off and my daughter came running over to where the tunnel area was and she said ‘Mummy, what's a dyke'. She was only about 10 or 11 at the time and I said ‘why honey?' and she said ‘Because that man over there, he has been calling you one the whole game' (Anne, referee).

You still do hear it from the crowd. ‘Get back in the kitchen' and derogatory comments talking about your body parts and that kind of thing (Jane, referee).

One of the worst stories I've heard was from a friend of mine who has actually quit refereeing now. A manager didn't think a decision she made was correct, so he told her ‘to go home and cook a roast dinner' (Dee, referee).

Many of the above examples reflect the exclusionary language identified by Sawiuk et al. (2021) as a significant determinant of women's levels of acceptance in football coach education environments. Whilst these comments could be readily dismissed as an innocuous part of the social dynamics of football, the accounts above highlight the insidious nature of misogynistic language. Here, lazy stereotypical “banter” that resorts to outdated connexions with traditional gender roles is readily interspersed with deeply threatening remarks underpinned by sexual or violent undertones. This is particularly alarming in light of research into sexual harassment and abuse affecting women in sport. Lewis et al.'s (2020) account of gender-based violence in coach education, for instance, highlights one example of how sexual innuendo, leering, and derogatory comments about the appearance of a woman coach acted as a precursor to physical sexual abuse. Although it may not necessarily be the case that all verbal remarks lead to violence, this does not diminish the threat that this poses for the recipients (Fasting and Brackenridge, 2009).

Perhaps more worryingly, many of the women identified that much of the abuse they received was just as prevalent in competitive youth [boys'] football as it was in the senior [men's] leagues.

I never had any sexist comments from the female players, but male players can often have something to say. One time I stopped the game for a foul and a 12 year old boy said ‘This is why girls should stick to refereeing girls' football' (Clare, referee).

First U16s match I reffed, I maybe did feel out my depth'cause I was just so nervous. Looking back now I would handle it completely differently. I had a boy - who I later found out was an actual referee – swearing, ‘this is why fucking women shouldn't referee'. I was 15 at the time. I didn't handle it very well and really didn't want to referee again after that match (Sarah, referee).

This was explained by some as resulting from the perception of women as a threat to the control that men hold over the game. Anya, for example, specifically commented that “the male coach felt threatened by me.”

Another prevalent response from men was the way in which some appeared to compliment or congratulate women on their performance within their role.

The reaction if you put on a decent session, it's almost an over-reaction sometimes and it becomes a little bit patronising. Erm, if you was a bloke and delivered that session I'm not sure they'd have raved about it as much as what they had, but because you're a female and maybe their expectations weren't as high as what it would have been if you were a male (Helen, coach).

There was some remarks about my skills and some banter about ‘haa, she's done you' when getting past the blokes in practise drills, which was a bit patronising (Millie, coach).

This could be interpreted as a positive step in which women are being acknowledged and rewarded for their credentials as coaches and referees, which in turn may signify a greater move toward male acceptance of women in football. Alternatively, we would argue that the condescending nature of the men's reactions depicted in these accounts is further testament to women's perceived incompatibility with coaching and refereeing (Schlesinger and Weigelt-Schlesinger, 2012). The accounts above demonstrate that the default assumption is that women are unskilled in football and lack the knowledge or ability to perform well in coaching or refereeing, and that when women demonstrate evidence to counter this belief, they are perceived as merely an exception to this standard expectation.

## “Just Grin and Bear It”—Coping Strategies For Remaining in the Game

The previous two themes bring to the fore the various challenges these women face in securing refereeing and coaching positions in football. The women's accounts also highlight a myriad of ways in which they mobilised their agency to circumvent these challenges. To help them negotiate their minority status and positioning as different, the women had developed a variety of coping strategies. Like Forbes et al. (2015), the first relates to an apparent denial or downplaying of sexism in the game. During interviews, a number of the participants were quick to dismiss the presence of gender-based discrimination in football coaching and refereeing. When asked if she had ever encountered any challenges as one of a minority of women in her profession, Hattie stated that she had “always just been viewed as a referee.” She further elaborated that “I've had a bad game and bad comments but never heard comments relating to being female.” Abi (referee) expressed a similar outlook:

Personally, I have not faced any barriers in the game. If you are good enough and have that bit of luck you will get to the next level.

At face value these accounts indicate positive change with respect to women's acceptance in coaching and refereeing roles (Jones and Edwards, 2013). However, what was most striking about the women's views was their failure to recognise experiences that might constitute sex discrimination that they had discussed at other points during their interview. For example, Hattie later acknowledged her minority status as a woman in refereeing, and discussed the efforts that were required for her to gain experience.

I do actually live in [town] but I ended up having to travel to [city] and referee in a junior league as they were the only league that would accept me.

Whilst Abi had not encountered any structural barriers to refereeing, she did acknowledge the presence of verbal sexist comments in the game. Rather than interpreting this as a problem, she argued that “this is part of football so I would not class it as a barrier.” A second coping mechanism therefore appeared to be their acceptance of sexism.

The fine line between “banter” and discrimination in sport is widely debated (Burdsey, 2011), and appears to be a particularly contentious issue in football (Cashmore and Cleland, 2011; Magrath et al., 2015). As highlighted earlier in the paper, sexist comments directed toward these women in their capacities as coaches and referees were commonplace, and whilst some were undoubtedly malicious and threatening, others were dismissed by the women, in Abi's words, as “part of the game.” Participants' narratives revealed the difficult position they were in regarding their response to these comments. For some there was a perception that it was something they had to accept if they wanted to progress within the game.

It's part of the job really, just learn to ignore it. Sometimes I feel some people are just wanting a reaction (Clare, referee).

Until there's more women in the environment, I don't think that's gonna change. Like I don't think education, educating young men not to speak like this would work. And to be honest I didn't really mind sometimes. You just brush it off, like ‘whatever'. So yeah, I think it kind of is, in a way, something that you've just got to just grin and bear (Ellie, coach).

Anne offered a similar insight. Her account indicated that there was a feeling among the older women who were among the first to qualify as referees that abuse had to be tolerated in order for them to survive within the game. Yet she also alludes to feelings of regret that she had not felt able to challenge discrimination.

I just let it go over my head really. I have made this comment over the years and wondered if our tolerance levels were too high. Maybe we could have made it easier for girls coming after us … but we were so keen to be accepted as referees and not women that perhaps we didn't challenge stuff that we should have (Anne, referee).

This illustrates the impossible position that many women find themselves in when working in male-dominated environments. Women must assimilate to survive, but in doing so they risk enabling the very discourses that constrain their inclusion to flourish.

For others, humour was used as a strategy to counter negative comments.

I'm the kind of person to laugh it off. I will probably use it in a game as a joke but I never take it too far, the players always know I'm joking. If I make a comment like ‘oh who would let females into the game' and keep it light hearted, then it's better. Especially in a women's game, we all have a laugh about it ‘cause they understand too (Hattie, referee).

Despite the women's acceptance of these comments, there was still an underlying sense of unease about their presence within the game.

A lot of men do not make comments because they want to be disrespectful, they do it sometimes because they don't know it's wrong. They call it banter, and I love banter and I don't think we should ever take it out of football but there comes a point when banter becomes something that touches a nerve with someone on the receiving end (Anne, referee).

The accounts of these women raise a number of points about the insidious and often uncontested nature of gendered power relations that pervade the context of football (Clarkson et al., 2019). It is useful to consider the work of Fink (2016, p. 2) here, in conceptualising the dominant gender discourse of wider sport. Fink (2016) argues that sport occupies a unique position in society with respect to the treatment of women. Whilst sexism in wider contemporary society is undoubtedly ever present, albeit in increasingly subtle ways, Fink (2016, p. 2) states that in sport, sexism is “commonly overt yet simultaneously unnoticed. It hides in plain sight. It is so entwined in the fabric of sport that most do not even discern it.” In football, in the context of England at least, it appears this may be particularly evident. Examples of the women's internalisation of their difference from men that were presented in the previous section, coupled with accounts highlighting their denial of sexism presented above are testament to this.

As a result of the pervasiveness of discourses of gender difference that circulated within the football context, a third coping strategy employed by the women involved engaging in a constant process of identity management. A number of the women were clear that when coaching and refereeing they were first and foremost a coach or referee: “I'm not a female referee—I'm a referee” (Jane). They wanted this identity to be reflected through their experiences within football. This meant that the women became skilful at reflecting on how they should present themselves at particular moments and in different places. They became adept at navigating the precarious process of identity management in order to find identity stasis—an equilibrium between their gender and footballing identities.

They would also expect me to turn up big, butch and masculine and I wasn't. Then again, I can't turn up too girly, ‘femininess', so you had to be careful what size heels you wear, how long is your skirt, how much neckline are you showing. All things that men don't even have to consider. They put their shirt and tie on and that's it. That's the sort of area that I found was always a challenge (Anne, referee).

I know once I get started I can use my voice and signals to assert my authority but I find it trickiest when I get there. I don't wear any makeup or anything so I probably look 12 years old and a small girl and they are probably thinking what on earth is going to happen (Sarah, referee).

Discussion of these processes of identity management highlighted the complexities and contradictions of the women's location within the football sphere. Their narratives echoed the double bind described by Forbes et al. (2015); they needed to appear “un-feminine” enough to convey legitimate and credible football expertise, yet simultaneously feminine enough to avoid succumbing to stereotypical homophobic discrimination that equates entry into male-defined terrain with transgression of the boundaries of “appropriate” gender and sexual identity. At the same time, as Anne's example attests, any display of conventional femininity in football events off the pitch is underscored with a perceived need to avoid displaying signs that could be open to the sexualised gaze of men. These concerns may be particularly apparent for these women in light of the prevalence of derogatory, threatening and often sexually suggestive remarks they face in their on-pitch roles, as we have outlined in the previous section. This again points toward women's tacit awareness of the threat of gender based violence in the context of male-dominated sport (Fasting and Brackenridge, 2009; Lewis et al.'s, 2020).

A final coping strategy the women drew on to ensure that they could maintain a working relationship with football related to managing their football career expectations. Many of the women were under no illusions that any aspirations to work in football, whether this be a career in the professional game or a paid position at lower levels, would be challenging to achieve.

You're going to go against a man in any job interview, your experience is never going to be as good as a man's … I don't feel like many women would be able to get into elite football (Zoe, coach).

I'm not sure that there is enough money in coaching, and what I mean by that is that I've got a house and a mortgage and I'm not sure that coaching, unless it was with a boys' academy, includes a liveable wage (Phoebe, coach).

Whilst the women were sceptical about the prospect of attaining a full-time career in coaching or refereeing, they were quick to assert that it was the enjoyment and satisfaction in these roles that enabled them to remain in the game. This meant that for most of the participants, they accepted that their involvement in football would most likely remain a hobby, and something they were prepared to undertake voluntarily or in a part-time paid capacity around other full-time jobs.

I imagine it will be that I will kind of work in centres of excellence or academies or, whatever, as a part-time thing alongside my full-time job which is obviously sport and coaching related but it's not kind of hands-on football coaching (Lauren, coach).

I work part-time for the family business doing the accounts and I also have two children so it is a bit of a juggle but I'm lucky that I work for my father-in-law and he is flexible in when I can work so I can tailor it around football (Jane, referee).

Others remained undeterred by the apparent lack of opportunities and had strategically sought additional (unpaid) avenues to make themselves more employable in the future. These women were also keen to attribute any success they had experienced so far in the game to their own tenacity, expertise, and hard work in the face of the various structural and discursive barriers they were presented with, rather than to any advances in policies promoting opportunities for women.

I think every opportunity I've probably created for myself rather than been given it, or, or applied for a job and got it sort of thing. I think I've been a pest and pestered people like ‘can I coach? Can I do this? Can I do that?' And from there I've been given the opportunities and then maybe made it into something else … and eventually got employed... so yeah, I've created opportunities, but it's been off my own back (Anya, coach).

Everything that I have created now I'd say I've done it through my own doing, going out my way and looking for jobs, looking for opportunities, getting onto courses, getting qualified, doing the CPD hours, doing everything that I can do to know that I've got the knowledge to do a job (Ella, coach).

In this respect, there is a need to consider the ways in which such opportunities are promoted and made accessible to women such that football coaching and refereeing become viable options for all women, rather than, as Anne put it, “just strong-minded stubborn women who go through with it anyway despite everyone telling us we can't.” Underlying each of the women's accounts of their football career trajectories was the acknowledgment that it was down to them to seek and negotiate opportunities for progression. This provides further testimony to the argument that meaningful institutional support for the development of gender equity in football is still lacking at structural levels within the game, and greater accountability is needed on the part of governing bodies and organisations (Welford, 2011; Jones and Edwards, 2013; Sawiuk et al., 2021).

## Concluding Remarks

The headlines at the beginning of this paper paint a rather bleak picture of women's experiences of football. At one level, it could be argued that these headlines merely sensationalise isolated incidences and should be read as nothing more. Indeed, the growth of football opportunities in school and community settings demonstrates not only an appetite to extend provision but also a desire on the part of thousands of women and girls to take up football. Some may use this evidence of growth to debunk the claims made by these headlines. Whilst we are heartened by these increasing trends in participation, we also concur with Caudwell (2011) that there remains a need to continue to critically explore the gendered nature of football and how this impacts those women invested in the game. In relation to women playing football, research has made considerable inroads in identifying that marginalisation, sexism, exclusion, and discrimination remain central tropes to the relationship between women and football. Our paper departs from a concern with *playing* the game and instead explores the experiences of women involved in football as coaches and referees. Within these leadership and management positions women continue to be underrepresented.

There is no doubt that the women involved in this research had enjoyable and rewarding coaching and refereeing experiences, with many having aspirations for further development and progression into the higher levels of the game. Yet the women were very much aware that they occupy a marginal position in an otherwise male dominated arena that has historically conspired to exclude them. Within this environment, their difference was continuously reinforced, resulting in constant comparisons with male norms, which positioned them as inferior within the game. This occurred in different ways, from repeated examples of condescending praise of their achievements, to much more threatening sexist and homophobic verbal abuse. The women in this research maintained their involvement in football by adopting a range of coping strategies. These included the denial or acceptance of gender-based discrimination, careful negotiation of their footballer and feminine identities, and management of their expectations around future development opportunities in the game. Despite their experiences, they expressed resilience and determination to challenge the established norms of the game and to strive for positive outcomes for other women in powerful football roles. However, an important finding to reiterate here is that underlying most of the women's accounts of their experiences in the game, was the irrefutable influence of their own individual agency in defining their success as referees or coaches. Very few referred to examples of club or governing body support, and where this was the case, it was not without criticism. In this sense, the women themselves were the greatest proponents of change in an industry that otherwise remains a bastion of hegemonic masculinity. Yet we must recognise that the women's ability to assert their agency is no doubt contingent on the presence of other aspects of their identity that hold privilege. For instance, the accounts highlight that their ability to succeed in football was heavily dependent on their ability to “perform” in ways that are normatively expected of footballers. Demonstration of appropriate levels of physical football skills appeared to be part of this and, in this sense, we might question whether non-disabled women might be better placed to be accepted as football coaches or referees than their disabled counterparts. Similarly, given the lesser likelihood of women securing paid coaching or refereeing opportunities than men, their social class background may be highly significant in determining whether they are able to make sacrifices to continue their pursuit of coaching and refereeing careers. In this respect, further research is needed to unpack the influence of intersecting aspects of women's identities that may affect their ability to succeed in football leadership roles.

Our presentation and discussion of the research findings highlights the necessity of adopting a theoretical approach that acknowledges how women can be situated in particular discursive configurations imbued with relations of power, whilst being active agents in challenging dominant discourses. Our findings highlight how women's football coach and referee identities are constituted within an ongoing process of negotiation between structure and agency. Their presence in football represents an ideological threat to the male control of the game, particularly when they infiltrate men's football spaces. It is no wonder, therefore, that opposition and abuse continue to flourish. We have no doubt that the institution of football, that is, governing bodies, leagues and clubs are making some concerted efforts to redress the gender inequalities, including for women coaches and referees. We draw confidence from all of this work but are also mindful that there is an enduring legacy of male control within football that stifles the possibilities for real change. Whilst awareness of these structural and experiential inequalities has triggered a range of policy and programme developments, there continues to be a need to honestly scrutinise these kinds of developments. In doing so, those responsible for the governance of football need to be bolder about the cultural shifts needed to spearhead lasting change.

## Data Availability Statement

The original contributions presented in the study are included in the article. Further inquiries can be directed to the corresponding author.

## Ethics Statement

The studies involving human participants were reviewed and approved by Leeds Beckett University. The participants provided their written informed consent to participate in this study.

## Author Contributions

SD supervised Projects 2 and 3. AS supervised Project 1. NH-A (Project 3), LP (Project 2), and JW-S (Project 1) were responsible for data collection. SD, AS, and HF conducted data analysis and interpretation and were collectively responsible for writing the manuscript. All authors contributed to the article and approved the submitted version.

## Funding

This work was supported by Leeds Beckett University.

## Conflict of Interest

The authors declare that the research was conducted in the absence of any commercial or financial relationships that could be construed as a potential conflict of interest.

## Publisher's Note

All claims expressed in this article are solely those of the authors and do not necessarily represent those of their affiliated organizations, or those of the publisher, the editors and the reviewers. Any product that may be evaluated in this article, or claim that may be made by its manufacturer, is not guaranteed or endorsed by the publisher.

## Footnotes

^1^This is a national scheme that aims to provide girls with positive opportunities to experience football in school through a network of 300 school partnerships, each having a dedicated expert with knowledge of girls' football in the local area. The ultimate goal is to create the next generation of active and confident footballers, coaches, officials, and leaders.

^2^This initiative takes place in primary schools and aims to inspire girls through Disney stories and imaginative play to become more active and develop fundamental football skills, and speaking and listening skills.

^3^These centres form part of a national network aiming to increase 5–11 year old girls' participation in football. The emphasis is on having fun, making friends, and developing physical skills to encourage a healthy lifestyle and develop a lifelong relationship with the game.

^4^The projects were granted ethical approval by the Leeds Beckett University Research Ethics Committee.

^5^Detailed information pertaining to each participant (including age, levels of experience, years of experience, county working within) is withheld purposefully in this paper. Such are the low levels of women operating at the more elite levels of the game, providing this detail could potentially compromise their anonymity.
